# Response Mechanism of cbbM Carbon Sequestration Microbial Community Characteristics in Different Wetland Types in Qinghai Lake

**DOI:** 10.3390/biology13050333

**Published:** 2024-05-10

**Authors:** Ni Zhang, Kelong Chen, Xinye Wang, Wei Ji, Ziwei Yang, Xia Wang, Junmin Li

**Affiliations:** 1 Qinghai Province Key Laboratory of Physical Geography and Environmental Process, College of Geographical Science, Qinghai Normal University, Xining 810008, China; zhangni0224@163.com (N.Z.); 202047341016@stu.qhnu.edu.cn (X.W.); jiwei100500@163.com (W.J.); 15756789182@163.com (Z.Y.); wx_813113@163.com (X.W.); 2 Key Laboratory of Tibetan Plateau Land Surface Processes and Ecological Conservation (Ministry of Education), Qinghai Normal University, Xining 810008, China; 3 National Positioning Observation and Research Station of Qinghai Lake Wetland Ecosystem in Qinghai, National Forestry and Grassland Administration, Haibei 812300, China; 4 School of Agricultural Engineering and Food Science, Shandong University of Technology, Zibo 255000, China; lijunmin@163.com

**Keywords:** Qinghai–Tibet Plateau, climate change, carbon cycle, carbon sequestration microorganisms, carbon fixation

## Abstract

**Simple Summary:**

In this paper, the differences in carbon sequestration microbial communities in different wetland types and their main influencing factors were investigated. It was found that the alpha diversity of cbbM carbon-sequestering microorganisms was consistent with the change trend in the total carbon content. *Acidithiobacillus* was used as a biomarker in lakeside wetlands, and *Thiothrix* and *Thiodictyon* were used as biomarkers in marsh wetlands. The diversity of cbbM carbon-fixing microorganisms was primarily influenced by the total nitrogen content, while the community structure was significantly affected by the soil total carbon content. The increase in soil temperature and humidity was conducive to the carbon-sequestering process of *Thiomicrospira*, *Thiomonas*, *Polaromonas* and *Acidithiobacillus*. The changes in wetland types seriously affected the characteristics of cbbM carbon sequestration in microbial communities, and a warm and humid climate may be conducive to wetland carbon sequestration.

**Abstract:**

Carbon-sequestering microorganisms play an important role in the carbon cycle of wetland ecosystems. However, the response mechanism of carbon-sequestering microbial communities to wetland type changes and their relationship with soil carbon remain unclear. To explore these differences and identify the main influencing factors, this study selected marsh wetlands, river wetlands and lakeside wetlands around Qinghai Lake as research subjects. High-throughput sequencing was employed to analyze the functional gene cbbM of carbon-sequestering microorganisms. The results revealed that the alpha diversity of cbbM carbon-sequestering microorganisms mirrored the trend in total carbon content, with the highest diversity observed in marsh wetlands and the lowest in lakeside wetlands. The dominant bacterial phylum was Proteobacteria, with prevalent genera including *Thiothrix*, *Acidithiobacillus*, and *Thiodictyon*. *Acidithiobacillus* served as a biomarker in lakeside wetlands, while two other genera were indicative of marsh wetlands. The hierarchical partitioning analysis indicated that the diversity of cbbM carbon-fixing microorganisms was primarily influenced by the total nitrogen content, while the community structure was significantly affected by the soil total carbon content. Moreover, an increased soil temperature and humidity were found to favor the carbon fixation processes of *Thiomicrospira*, *Thiomonas*, *Polaromonas*, and *Acidithiobacillus*. In summary, changes in wetland types seriously affected the characteristics of cbbM carbon sequestration in microbial communities, and a warm and humid climate may be conducive to wetland carbon sequestration.

## 1. Introduction

Soil is the largest terrestrial carbon reservoir, storing far more carbon than plants and the atmosphere [1,2]. Wetland soil carbon storage accounts for 1/3 of the total soil carbon storage on land and has great potential for regulating atmospheric carbon dioxide concentrations and mitigating climate change [3,4]. Therefore, wetlands are extremely important in the regulation of the global carbon balance of terrestrial ecosystems [5,6,7]. Maintaining a high carbon storage in wetland ecosystems also plays an important role in mitigating climate warming caused by increasing carbon dioxide (CO_2_) concentrations [8,9]. However, climate change also affects the ability of wetlands to sequester carbon [10,11]. In the foreseeable future, global temperatures will continue to rise [12], and the frequency and intensity of biogeochemical cycles will further increase [13]. These changes may exacerbate land degradation processes, have strong impacts on ecosystem functions and biological interactions [14,15], and they may even significantly affect the carbon sequestration capacity of wetlands. Previous studies have shown that different wetland types can lead to changes in vegetation types and further lead to changes in the size of the organic carbon pool and its chemical composition [16,17,18]. Currently, published carbon sequestration rates for various wetlands range from 0.02 to 6 Mg SOC ha^−1^-year^−1^, and this difference is also closely related to the wetland type [19]. Therefore, it is necessary to study the carbon fixation mechanism of different wetland types.

As the “engine” of the biogeochemical cycle, microorganisms usually drive the carbon cycle of wetland soil through catabolism and anabolism [2,20]. Carbon sequestration microorganisms are critical to the conservation and restoration of the carbon sequestration potential of wetland soils and soil functions, and they act by absorbing carbon dioxide and converting atmospheric CO_2_ into organic carbon [21]. Carbon sequestration microbial groups fix CO_2_ through six main pathways [22,23,24]. The Calvin cycle is the most important CO_2_ fixation pathway for carbon sequestration microorganisms, and the key enzyme involved in this cycle is 1,5-diphosphate ribulose carboxylase/oxygenase (RubisCO) [25]. Two functional genes, cbbL and cbbM, are highly conserved and encode large subunits of RubisCO forms I and II, respectively, and they are commonly used as biomarkers to measure carbon sequestration in the environment [26]. However, Liu et al. [27] investigated the controlling factors and driving microorganisms of dark carbon fixation in intertidal sediments and found that cbbM-carrying bacteria were more responsible for carbon sequestration in ecosystems than cbbL-carrying bacteria were, confirming the importance of cbbM functional genes.

With an average elevation of more than 4000 m, the Qinghai–Tibet Plateau has the largest area of alpine wetlands in the world [28], and was also the first region affected by climate change in China [29]. Global climate change has had a significant impact on the carbon cycle of the Qinghai–Tibet Plateau ecosystem [30]. Recent studies found that climate warming will cause changes in various hydrological processes in the Qinghai–Tibet Plateau water system, which may adversely affect its ecological structure, function and resilience [31,32]. Therefore, in this study, the Qinghai Lake Basin in the northeastern Qinghai–Tibet Plateau was selected as the research area, and the riverhead wetlands, lakeside wetlands and swamp wetlands in the Qinghai Lake Basin were selected as research objects. High-throughput sequencing technology was used to determine the microflora of cbbM functional genes, and the biogeochemical properties of the soil were also determined. The objectives of this study were to (1) study the response patterns of cbbM carbon sequestration microbial communities to different wetland types; (2) evaluate the effects of soil properties driven by different wetland types on cbbM carbon sequestration microbial communities; and (3) analyze the interaction between cbbM carbon sequestration microorganisms and environmental factors in different wetland types in the Qinghai Lake Basin. The results can not only provide basic data for the quantitative study of the carbon cycle and transformation in the Qinghai Lake Basin but also provide a reference and guidance for the study of the mechanism of carbon sources and sinks in alpine wetlands.

## 2. Materials and Methods

### 2.1. Overview of the Study Area

Wayan Mountain, situated between 37°43′ and 37°46′ N and 100°01′ and 100°05′ E, is a characteristic riverhead wetland. It boasts an elevation ranging from 3720 to 3850 m, an annual mean temperature of −3.31 °C, and an average annual precipitation of 420.37 mm. The vegetation here is primarily dominated by *Kobresia humilis (C. A. Mey. ex Trautv.) Serg*. Xiaobo Lake, on the other hand, is a year-round flooded swamp wetland with coordinates of 36°41′ to 36°42′ N and 100°46′ to 100°47′ E. It has an average elevation of 3228 m, an annual mean temperature ranging from −0.8 to 1.1 °C, and an average annual precipitation of 324.5 to 412.8 mm. The wetland’s flora is primarily composed of *Kobresia humilis (C. A. Mey. ex Trautv.) Serg* and *Blysmus sinocompressus Tang et Wang*. Bird Island, located between 36°57′ and 37°04′ N and 99°44′ and 99°54′ E, is a typical lakeside wetland. It has an elevation of 3194 to 3226 m, an average annual temperature of −0.7 °C, and an average annual precipitation of 322.7 mm. The dominant species found in this wetland type are *Allium przewalskianum Regel*, *Astragalus adsurgens Pall*, and *Poa annua L* [33].

### 2.2. Soil Sample Collection

In June 2020, during the early stage of plant growth, soil samples were collected. Each plot was 1 m × 1 m in size, and a five-point sampling method was used to collect soil from the 0–10 cm surface layer using a soil auger with a diameter of 4.5 cm. The samples were named according to the experimental station name as Wck (Wayan Mountain), Bck (Xiaobo Lake), and Nck (Bird Island). Five replicates were collected at each sampling site, resulting in a total of 15 soil samples. These samples were mixed and sieved through a 2 mm mesh sieve. Some soil samples were preserved in liquid nitrogen tanks for soil DNA extraction, while the remaining samples were stored in ice bags for rapid transportation back to the laboratory for further analysis.

### 2.3. Determination of Soil Physical and Chemical Properties

A TDR-300 (produced by Spectrum Technologies in Plainfield, IL, USA) is utilized to monitor soil moisture levels within a 0–10 cm depth. Meanwhile, the LI-8100 instrument (manufactured by LI-COR in Lincoln, NE, USA) measured the soil temperature within the same depth range. For pH measurements, a pH meter (model FE20-FiveEasy pH, from Mettler Toledo in Gießen, Germany) was employed after mixing the soil with water at a ratio of 1:2.5. To determine total carbon (TC) and total nitrogen (TN) content, an Elemental Analysis System (Vario EL III, Elemental Analysis System GmbH, Langenselbold, Germany) was used [34].

### 2.4. DNA Extraction and Illumina MiSeq Sequencing

Soil microbial DNA was extracted from 0.5 g of fresh soil using a PowerSoil DNA Isolation Kit (Mio-bio, Carlsbad, CA, USA). Standard fixed carbon microbial amplification primers, namely the forward primer (5′-TTCTGGCTGGGBGGHGAYTTYATYAARAAYGACGA-3′) and the reverse primer (5′-CCGTGRCCRGCVCGRTGGTARTG-3′), were used to amplify the cbbM gene fragment [35]. The Illumina MiSeq sequencing platform was utilized to sequence the PCR products obtained. DNA extraction, quantification, and PCR procedures were carried out following previously established and validated protocols [35]. This approach ensured the accuracy and reproducibility of the sequencing results.

### 2.5. Statistical Analysis

Functional groups of microorganisms were predicted by FAPROTAX [36]. Using R software (version 4.1.2), the *p*-value was calculated and plots were generated, referencing specific R packages and functions from paper [34].

## 3. Results

### 3.1. Community Diversity of cbbM Carbon Sequestration Microorganisms in Different Wetland Types

**Figure 1:**
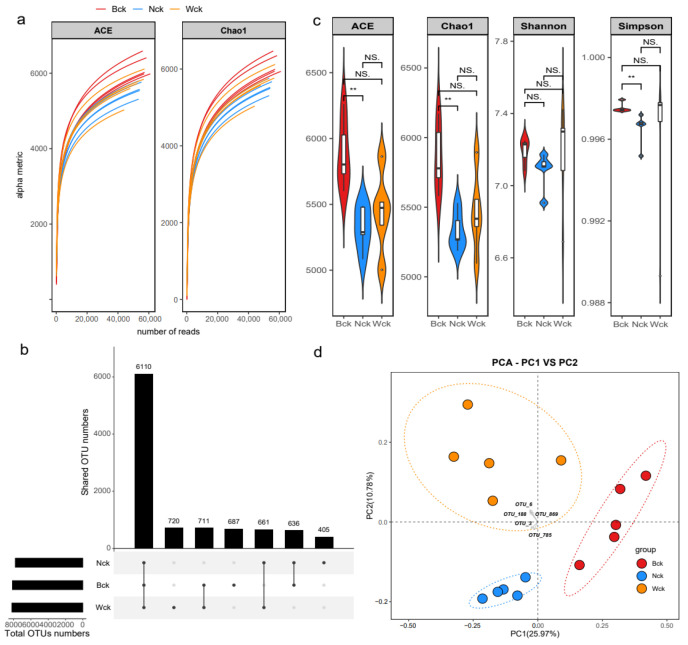
Illumina sequencing results and carbon sequestration microbial community diversity: (**a**) sample dilution curve; (**b**) OTU distribution map; (**c**) cbbM microbial alpha diversity index; (**d**) cbbM microbial principal component analysis. NS indicates *p* > 0.05, and ** indicates *p* < 0.01.

The sequencing results indicated that the partial dilution curve did not reach saturation (Figure 1a); it rather approached saturation, suggesting a comprehensive representation of the diversity of carbon-sequestering bacterial communities containing cbbM genes. Additionally, based on the calculation of Good’s coverage index (ranging from 0.9749 to 0.9826), higher coverage indices of the samples corresponded to smaller proportions of undetected species. According to Illumina MiSeq analysis, at a 3% sequence difference level clustering, the number of operational taxonomic units (OTUs) of cbbM carbon-sequestering microorganisms in Qinghai Lake wetlands was 9930 (Figure 1b). The OTU counts of marsh wetlands, lakefront wetlands and riverhead wetlands varied from 7812 to 8202, with unique OTUs of 687, 405, and 720, respectively. Notably, the alpha diversity varied among the different wetland types (Figure 1c). While the species richness and evenness indices of riverhead wetlands fell between those of marsh and lakeside wetlands, with no statistically significant differences, marsh wetlands exhibited significantly higher species richness and evenness indices compared to lakeside wetlands, highlighting a notable disparity between them. As shown in Figure 1d, a PCA based on the OTU levels illustrated distinct differences among samples from the three wetland types. Generally, lakeside wetlands exhibited minimal soil heterogeneity and similar community compositions of carbon-sequestering microorganisms. Conversely, river source wetlands displayed the greatest soil heterogeneity, with slightly larger differences in carbon sequestration microbial community composition among samples.

### 3.2. Composition of cbbM Carbon Sequestration Microbial Communities in Different Wetland Types

**Figure 2:**
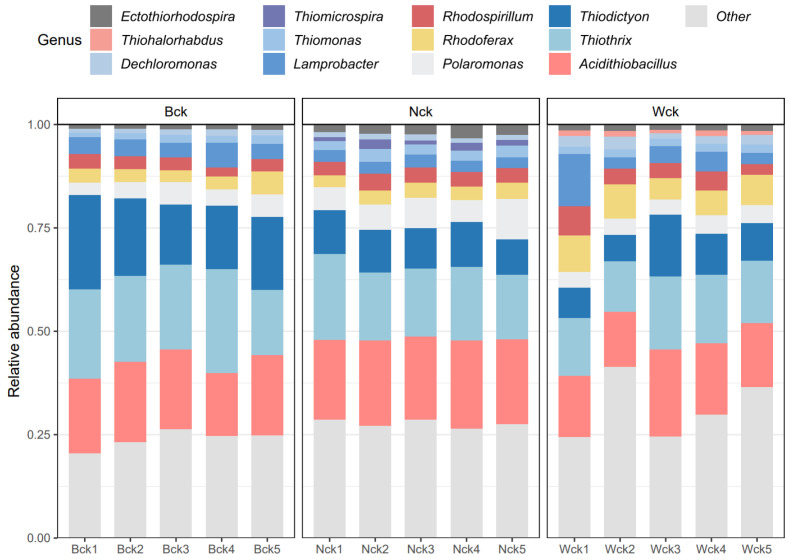
Community composition of cbbM carbon sequestration microorganisms in Qinghai Lake wetlands.

**Figure 3:**
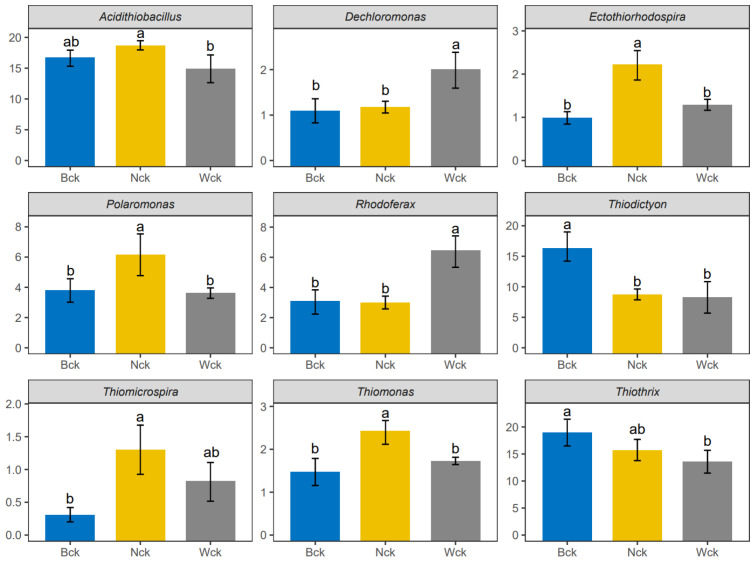
Genera-level difference of microflora of three wetland types in Qinghai Lake. abc indicates significance, the same letter indicates no significant difference between groups (*p* > 0.05), and different letters indicate a significant difference between groups (*p* < 0.05).

At the phylum level, proteobacteria emerged as the dominant bacterial group in the wetland soil of Qinghai Lake, constituting a relative abundance exceeding 99.9%. Unclassified genera accounted for 23.09% to 30.32% of bacterial relative abundance. Twelve genera-level bacteria with relative abundances greater than 1% in the Qinghai Lake wetland were selected to construct a histogram of relative abundance percentages (Figure 2). *Thiothrix*, *Acidithiobacillus* and *Thiodictyon* were the most abundant, all belonging to Proteobacteria, with average relative abundances of 17.18%, 17.75% and 12.01%, respectively. ANOVA analysis revealed that nine genera-level microflora (relative abundance > 1%) were significantly influenced by wetland type (Figure 3). Distinct biomarkers were identified for different wetland types. *Acidithiobacillus*, *Ectothiorhodospira*, *Polaromonas*, *Thiomicrospira* and *Thiomonas* exhibited the highest relative abundances in lakeside wetlands. *Dechloromonas* and *Rhodoferax* were most abundant in river source wetlands, while *Thiodictyon* and *Thiothrix* dominated in swamp wetlands.

### 3.3. Functional Groups of cbbM Carbon Sequestration Microbial Community in Qinghai Lake Wetlands

**Figure 4:**
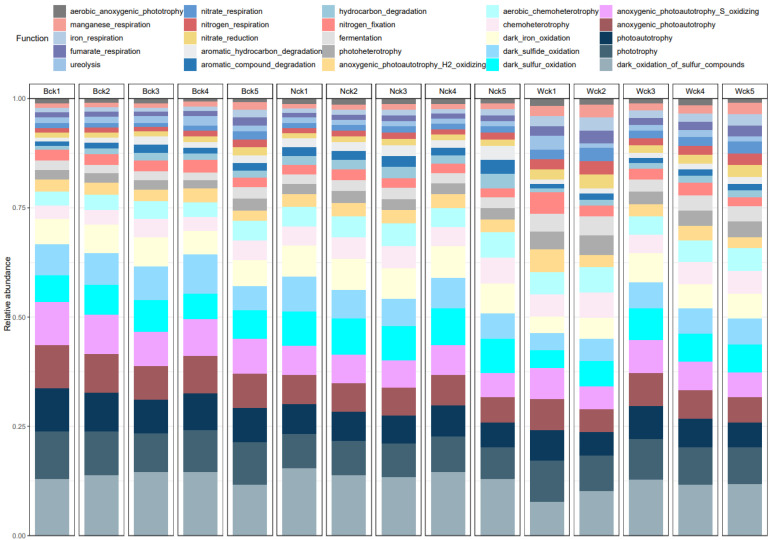
Main functional groups of cbbM carbon sequestration microorganisms in the Qinghai Lake wetlands.

**Figure 5:**
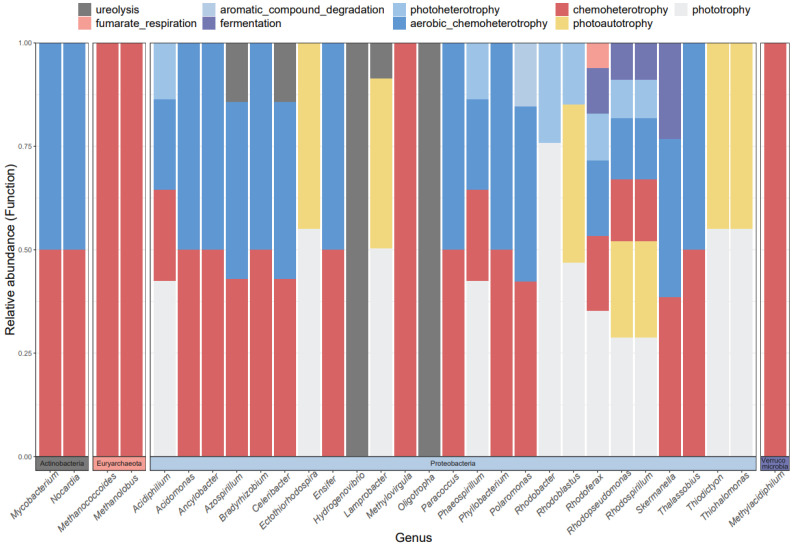
The main functional groups of the C cycle and the corresponding generic level microflora in the Qinghai Lake wetlands.

The FAPROTAX function annotation results of the carbon sequestration microbial community in Qinghai Lake wetlands (Figure 4) revealed that the ecological functions of the community could be categorized into 25 functional groups (with relative abundances exceeding 1%). Among the microbial functions associated with cbbM (Top 10), the predominant ones included dark_oxidation_of_odor_compounds (12.49%), phototrophy (8.71%), anoxygenic_photoautotrophy (7.13%), photoautotrophy (7.13%), anoxygen_photoautotrophy_S_oxidizing (7.13%), dark_oxidation (6.72%), dark_sulfide_oxidation (6.33%), dark_iron_oxidation (5.96%), chemoheterotrophy (4.53%), and aerobic_chemoheterotrophy (4.52%). The relative abundance of each was closely related to wetland type. The corresponding microflora were reversed through the nine main functional groups of the C cycle (Figure 5), and it was found that cbbM carbon sequestration microorganisms in the Qinghai Lake wetland were in 30 genus-level microflora of four phyla, of which 25 genus-level microflora belonged to Proteobacteria. The predominant functional groups among most bacteria were phototrophs and photoautotrophs, while some bacteria also exhibited chemoheterotrophs and aerobic chemoheterotrophs as primary functional groups.

### 3.4. Correlations between the cbbM Carbon Sequestration Microbial Community and Soil Environmental Factors in the Qinghai Lake Wetlands

**Figure 6:**
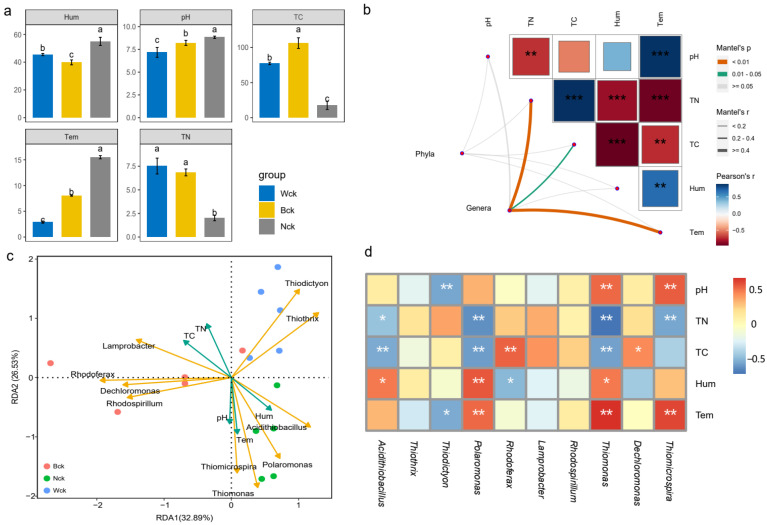
Correlation between soil environmental factors and carbon-sequestering microorganisms in the Qinghai Lake wetlands: (**a**) changes in physicochemical factors in different types of wetlands; (**b**) correlation network diagram between carbon-sequestering microbial community characteristics and environmental factors; (**c**) redundancy analysis of environmental factors and genus-level microflora (Top 10); (**d**) heatmap of correlation between environmental factors and genus-level microflora (Top 10). abc indicates significance, the same letter indicates no significant difference between groups (*p* > 0.05), and different letters indicate a significant difference between groups (*p* < 0.05); * indicates *p* < 0.05, ** indicates *p* < 0.01, *** indicates *p* < 0.001.

**Figure 7:**
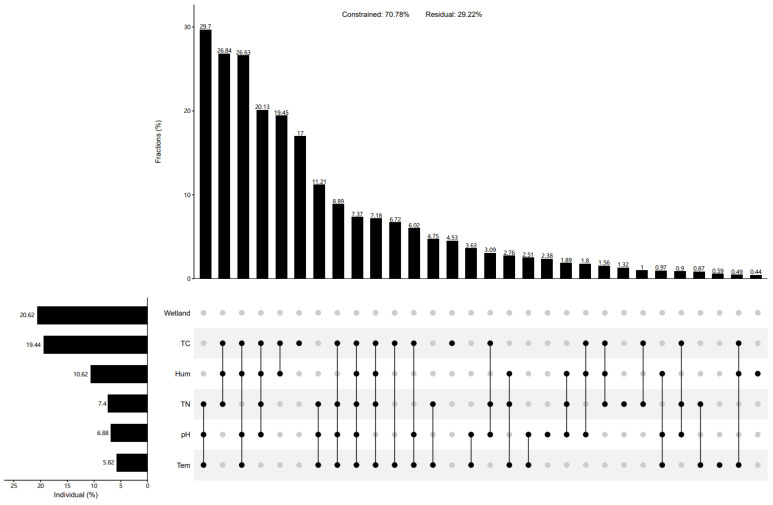
Hierarchical segmentation analysis of influencing factors of community structure.

**Figure 8:**
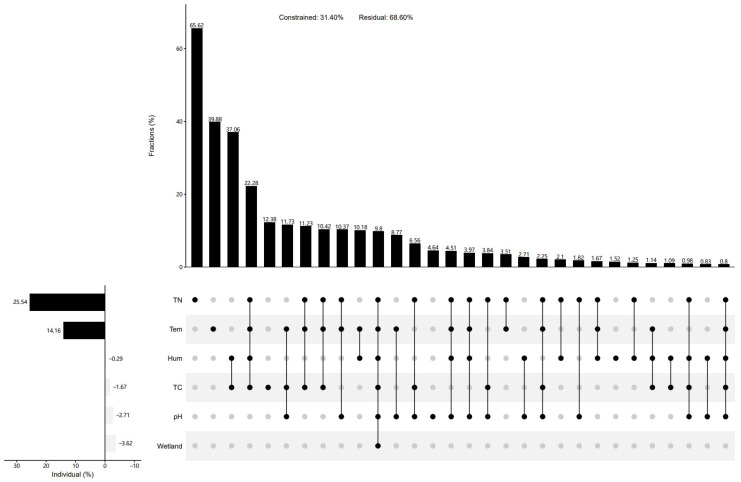
Hierarchical segmentation analysis of influencing factors of Alpha diversity.

The physical and chemical factors of the soil were significantly influenced by wetland types, displaying notable spatial variations (*p* < 0.05) (Figure 6a). Regarding physical factors, lakeside wetlands exhibited significantly higher temperatures and humidity levels compared to marsh and riverhead wetlands. Although the soil moisture of the riverhead wetland was higher than that of the marsh wetland, the soil temperature of the riverhead wetlands was lower than that of the marsh wetlands. The pH values of the Qinghai Lake wetlands followed a similar trend to soil temperature variations, while total carbon and nitrogen contents were lowest in lakeside wetlands. Additionally, marsh wetlands displayed a higher total carbon content than river source wetlands, with the trend reversed for total nitrogen content. Positive correlations were observed between soil temperature and moisture, as well as between soil total carbon and nitrogen contents (*p* < 0.05). However, no significant correlations were found between pH and total carbon content or soil moisture (*p* > 0.05), while other physical and chemical factors exhibited significant negative correlations (*p* < 0.05) (Figure 6b). At the phylum level, soil environmental factors did not significantly influence the community of carbon-fixing microorganisms (*p* > 0.05). However, at the genus level, microbial communities were closely correlated with soil temperature and total carbon and nitrogen content (*p* < 0.05) (Figure 6b). A redundancy analysis of the top 10 carbon sequestration microflora and soil environmental factors revealed that different environmental factors had varying impacts on different microorganisms. The pH exhibited minimal impact on carbon sequestration microbial communities. Further correlation analyses demonstrated significant positive correlations between pH and *Thiomicrospira* and *Thiomonas*, and significant negative correlation with *Thiodictyon*. Soil temperature and humidity showed positive correlations with Thiomicrospira, *Thiomonas*, *Polaromonas*, and *Acidithiobacillus*, while total carbon and nitrogen exhibited negative correlations with these microflora. Moreover, the total carbon content displayed significant positive correlations with Dechloromonas and Rhodoferax, potentially important markers of cbbM carbon sequestration in Qinghai Lake wetlands. Hierarchical partitioning analysis indicated that the wetland type, total carbon, and humidity explained the majority of variation in the community structure of cbbM carbon-fixing microorganisms in Qinghai Lake wetlands (Figure 7). The total carbon emerged as the most significant environmental factor, interacting with other factors to influence the assembly of wetland carbon-fixing microbial communities (Figure 7). While the alpha diversity of carbon-fixing microorganisms exhibited a less pronounced response to wetland type, it was primarily influenced by soil physicochemical properties, with total nitrogen being the primary driver, while temperature also played an important role (Figure 8).

## 4. Discussion

### 4.1. Effects of Wetland Type Changes on cbbM Carbon Sequestration Microbial Community Diversity

Richness and diversity serve as two crucial indicators of carbon sequestration microbial community characteristics, and they are significantly influenced by the heterogeneity of wetland types [37]. The richness and diversity of cbbM carbon sequestration microbial communities in the Qinghai Lake wetlands responded to changes in wetland types to a certain extent. The richness and diversity indices of the microbial community in marsh wetlands were significantly higher than those in lakeside wetlands. However, the difference between river source wetlands and the other two types of wetlands was not as pronounced, possibly due to the high carbon and nitrogen contents in marsh wetlands, which promote the activity of carbon-sequestering microorganisms [38,39]. The carbon and nitrogen contents of lakeside wetlands were also significantly lower than those of marsh wetlands, further supporting this view. Previous studies have indicated that the diversity of carbon sequestration microbial communities on the Qinghai–Tibet Plateau is closely related to environmental factors. Soil moisture and pH are generally regarded as key factors determining soil microbial diversity [40,41]. For instance, Hu [42] demonstrated a significant correlation between microbial diversity and variations in soil moisture, with the latter also exerting a notable influence on soil nutrient variations. Wang [43] conducted a study examining the influence of environmental factors on microbial communities, revealing that pH impacts these communities by modulating carbon and nitrogen content. Additionally, Wang [44] investigated the factors affecting the carbon sequestration microbial community under changes in precipitation on the Tibetan Plateau and found that the soil temperature, humidity, and pH were the most important factors influencing the diversity of the carbon sequestration microbial community. In Wang’s [45] research on the influencing factors of carbon sequestration microbial communities in the Tibetan Plateau, changes in total nitrogen content significantly affected carbon sequestration microorganisms. This study also identified the total nitrogen content as the most influential factor on the alpha diversity of carbon-fixing microbial communities in the Qinghai Lake wetlands, with the soil temperature also playing a significant role. The significant differences in total nitrogen content and temperature between lakeside wetlands and marsh wetlands also provide support for these findings. However, their correlation with pH and humidity was relatively weak, possibly due to small spatial scales and consistent land use practices [46,47].

### 4.2. Effects of Wetland Type Changes on cbbM Carbon Sequestration Microbial Community Structure

Proteobacteria were the dominant bacteria in the carbon sequestration microbial communities of the three types of wetlands in Qinghai Lake, consistent with the research findings of Wang et al. [48] on carbon sequestration microorganisms in karst wetlands. Similarly, Gao et al. [49] investigated the community characteristics of carbon sequestration microorganisms on the northern Tibetan Plateau and reached similar conclusions. However, numerous studies have shown that the community composition of cbbM carbon sequestration microorganisms in wetland ecosystems is different at the genus level. Wang et al. [48] investigated the abundance and diversity of carbon sequestration bacterial communities in karst wetland soil ecosystems. The dominant bacterial genera of cbbM carbon-sequestering microorganisms were *Ferriphaselus*, *Halothiobacillus*, *Rhodopseudomona*, *Sinorhizobium* and *Sulphitalea*. Yousuf et al. [50] compared cbbM carbon-sequestering microbial communities in saline soil and farmland soil and found that *Rhodopseudomonas* and *Thiobacillus* were the dominant bacterial genera in farmland soil. In this study, the dominant bacterial genera of cbbM carbon sequestration microorganisms in the Qinghai Lake wetlands were *Thiothrix*, *Acidithiobacillus* and *Thiodictyon*, which differed from previous studies. Yang et al. [51] investigated the dynamics of soil organic carbon and nitrogen in coastal wetlands in eastern China after Spartamina alterniflora invasion and found that the coastal salt marsh wetlands were in a local state of hypoxia, and this unique environment produced a unique dominant genus of carbon fixation microorganisms. Therefore, the differences in the dominant bacterial genera of carbon-sequestering microorganisms in wetland ecosystems are closely related to changes in the microenvironment. A correlation analysis between carbon-fixing microorganisms and soil physicochemical factors in Qinghai Lake wetlands indicated that the community structure of carbon-fixing microorganisms was primarily influenced by the soil total carbon content. Wang et al. [48] also found that changes in soil carbon components are the main factors influencing the structure of wetland soil carbon-fixing microbial communities, which is consistent with the results of this study. In addition, soil temperature and humidity were positively correlated with *Thiomicrospira*, *Thiomonas*, *Polaromonas* and *Acidithiobacillus*, while total carbon and nitrogen were negatively correlated with these four microbial communities, indicating that a higher soil temperature and humidity might be more conducive to the carbon sequestration process of these microbial communities. The dominant species of bacteria in the Qinghai Lake wetlands were significantly affected by the wetland types, and the relative abundance of *Acidithiobacillus* in lakeside wetlands was the highest, which may be due to the higher temperature in lakeside wetlands and the thermophilic characteristics of the bacteria [52]. The relative abundances of *Thiodictyon* and *Thiothrix* were the highest in swamp wetlands, which may be related to the high carbon content in this wetland type.

## 5. Conclusions

This study compared the characteristics of cbbM carbon sequestration microbial communities and their correlation with soil environmental factors in three types of wetlands in Qinghai Lake. The alpha diversity of the carbon sequestration microbial community was significantly different between marsh wetlands and lakeside wetlands, with the highest diversity in marsh wetlands, followed by riverhead wetlands and then lakeside wetlands. The dominant species composition of cbbM carbon-sequestering microorganisms in the three wetland types was similar, with Proteobacteria as the dominant bacterial group at the phylum level and *Thiothrix*, *Acidithiobacillus* and *Thiodictyon* as the dominant bacterial groups at the genus level. However, *Acidithiobacillus* had the highest relative abundance in lakeside wetlands, while *Thiothrix* and *Thiodictyon* had the highest relative abundance in marsh wetlands. Total nitrogen was the most significant influencing factor on the alpha diversity of soil carbon-fixing bacterial communities in Qinghai Lake wetlands, with the soil total carbon content being the primary soil physicochemical factor affecting community structure. The changes in wetland types result in variations in soil microenvironments and environmental factors. Marsh wetlands are more conducive to the carbon sequestration process in wetland ecosystems. This study provides a scientific basis and reference for soil carbon sequestration and ecological protection of alpine wetland ecosystems.

## Data Availability

Raw data have been uploaded to NCBI, and Its BioProject is PRJNA1006296.
